# Imaging for the diagnosis and response assessment of renal tumours

**DOI:** 10.1007/s00345-018-2342-3

**Published:** 2018-06-13

**Authors:** Sabrina H. Rossi, Davide Prezzi, Christian Kelly-Morland, Vicky Goh

**Affiliations:** 1 Academic Urology Group, University of Cambridge, Addenbrooke’s Hospital, Cambridge Biomedical Campus, Cambridge, CB2 0QQ UK; 2 Cancer Imaging, School of Biomedical Engineering & Imaging Sciences, King′s College London, London, UK; 3 Department of Radiology, Guy’s & St Thomas’ NHS Foundation Trust, London, SE1 7EH UK

**Keywords:** Renal cancer, Imaging, Diagnosis, Staging, Treatment response

## Abstract

**Purpose:**

Imaging plays a key role throughout the renal cell carcinoma (RCC) patient pathway, from diagnosis and staging of the disease, to the assessment of response to therapy. This review aims to summarise current knowledge with regard to imaging in the RCC patient pathway, highlighting recent advances and challenges.

**Methods:**

A literature review was performed using Medline. Particular focus was paid to RCC imaging in the diagnosis, staging and response assessment following therapy.

**Results:**

Characterisation of small renal masses (SRM) remains a diagnostic conundrum. Contrast-enhanced ultrasound (CEUS) has been increasingly applied in this field, as have emerging technologies such as multiparametric MRI, radiomics and molecular imaging with ^99m^technetium-sestamibi single photon emission computed tomography/CT. CT remains the first-line modality for staging of locoregional and suspected metastatic disease. Although the staging accuracy of CT is good, limitations in determining nodal status persist. Response assessment following ablative therapies remains challenging, as reduction in tumour size may not occur. The pattern of enhancement on CT may be a more reliable indicator of treatment success. CEUS may also have a role in monitoring response following ablation. Response assessments following anti-angiogenic and immunotherapies in advanced RCC is an evolving field, with a number of alternative response criteria being proposed. Tumour response patterns may vary between different immunotherapy agents and tumour types; thus, future response criteria modifications may be inevitable.

**Conclusion:**

The diagnosis and characterisation of SRM and response assessment following targeted therapy for advanced RCC are key challenges which warrant further research.

## Introduction

Over 337,000 new cases of renal cell carcinoma (RCC) are diagnosed annually worldwide [1]. In Western European countries such as the UK, the majority of renal cancers are early of stage (stage I–II: 56%), as a result of the rising incidence of incidentally detected tumours with the increased use of cross-sectional imaging [2, 3]. Characterisation of incidental renal lesions remains a diagnostic challenge, particularly for small renal masses (SRM, < 4 cm in size) and in differentiating malignancy from oncocytoma and fat-poor angiomyolipoma. Accurate diagnosis of incidental SRM has life-changing consequences for patients as well as economic implications [4]. Accurate staging of RCC and radiologic assessment of response to therapy are crucial to guide management and to deliver realistic information regarding treatment and prognosis to patients [5]. Imaging plays a key role throughout the RCC patient pathway, from diagnosis and staging of the disease, to the assessment of response to therapy. This review aims at summarising current knowledge regarding imaging as applied to the RCC patient pathway, highlighting advances and focusing on key challenges to guide further research. We therefore summarise current and emerging evidence regarding imaging for the diagnosis, staging and response assessment of renal cancer.

## Methods

A non-systematic literature search was conducted using Medline, updated to December 2017. For each of the sub-sections of the manuscript (diagnosis, staging and response assessment), a separate literature search was performed, using combinations of the following key words and medical subject headings: RCC, kidney cancer, renal carcinoma, renal cancer, small renal mass, Bosniak cyst, diagnosis, staging, response, ablation and metastatic. Each of the imaging modalities was also investigated in turn. The search was limited to English language studies. The reference lists of selected manuscripts were checked manually for eligible articles. The most relevant articles summarising existing knowledge of imaging in renal cancer were selected for this review. In addition, key international guidelines were reviewed in urology, radiology and oncology, including the European Association of Urology (EAU), American Urological Association (AUA), Canadian Urological Association (CUA), European Society for Medical Oncology (ESMO), American College of Radiologists (ACR) and the Cardiovascular and Interventional Radiological Society of Europe (CIRSE) [5–11]. Key criteria for response assessment were also manually searched [12–18].

## Results

### Diagnosis and characterisation

#### Symptomatic vs. asymptomatic patients

Symptomatic patients typically present with more advanced RCC than patients with incidentally detected tumours [19, 20]. The prevalence of RCC in patients presenting with visible haematuria is 0.9–2.0%; 0.3–1.0% in patients with microscopic haematuria [21, 22]. Computed tomography (CT) urography is recommended as the most appropriate first-line test for patients presenting with unexplained visible haematuria by the AUA and the CUA [9, 10]. However, there is a lack of consensus regarding the optimal investigation of asymptomatic microscopic haematuria in different guidelines worldwide in terms of imaging modality (ultrasound vs. CT urography) and age threshold to prompt investigation [23]. An ultrasound (US) of the renal tract may be considered initially in low-risk young patients with non-visible haematuria as a cost-effective non-ionising technique for assessing the kidneys and bladder [24, 25]. However, US sensitivity is low for renal lesions < 1 cm (26%) [26] and it does not evaluate the collecting systems adequately; hence, CT urography (pre- and post-contrast) is recommended as a first-line tool for patients presenting with non-visible haematuria by a number of American associations [9, 11, 23, 27]. CT urography following contrast administration assesses the entire urinary tract [28] and has a better diagnostic yield for renal cancer than intravenous urography (IVU), with a sensitivity of 100%, specificity of 97.4% and accuracy of 98.3% [29]. Magnetic resonance (MR) urography has the advantage of higher soft tissue contrast compared to CT and it has no radiation burden, but is prone to motion artefacts and limited by MR contraindications and scanner availability [30]. MR urography may be reserved for problem-solving, in pregnancy or when patients have an iodinated contrast allergy or renal failure.

Up to 50% of new cases of renal cancer will be detected incidentally in asymptomatic patients undergoing imaging for an unrelated indication [31]. In fact, over 40% of Medicare insurance beneficiaries in the USA undergo CT of the chest and abdomen over a 5-year period [32]. In a study of 3001 adults undergoing CT colonography, 14.4% of patients had at least one renal mass > 1 cm [33]. However, characterisation of incidentally detected small renal tumours remains a major challenge for imaging.

#### Cystic masses

**Fig. 1:**
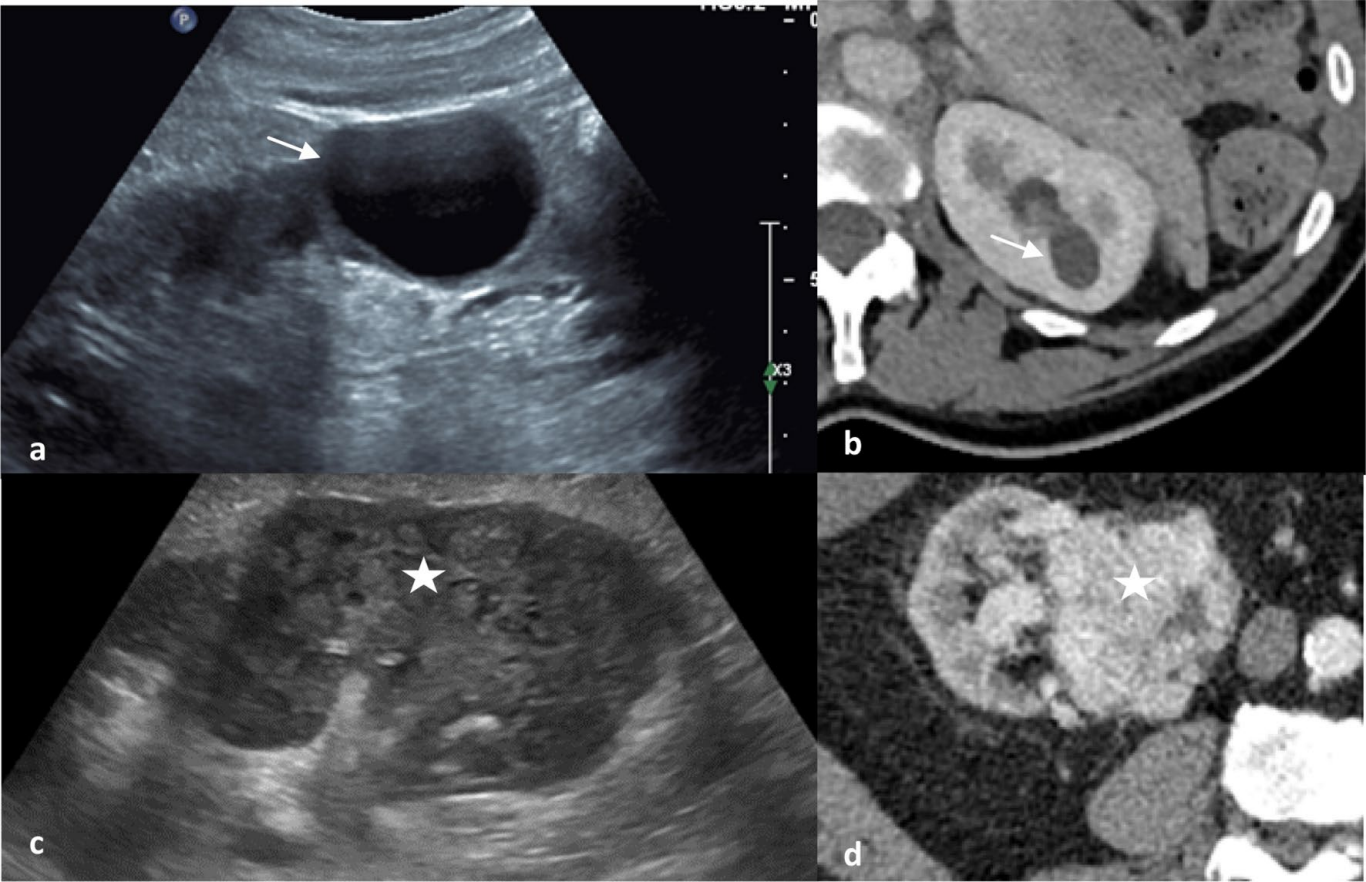
Ultrasound (**a**) and CT (**b**) appearances of a simple cyst with a thin imperceptible wall and posterior acoustic enhancement and no internal echoes or enhancement (Bosniak I). Contrast this to the ultrasound (**c**) and CT (**d**) features of a clear cell renal cell carcinoma with ill-defined borders and solid mixed echogenicity replacing renal parenchyma and avid contrast enhancement with areas of low attenuation tumoral necrosis on CT

**Fig. 2:**
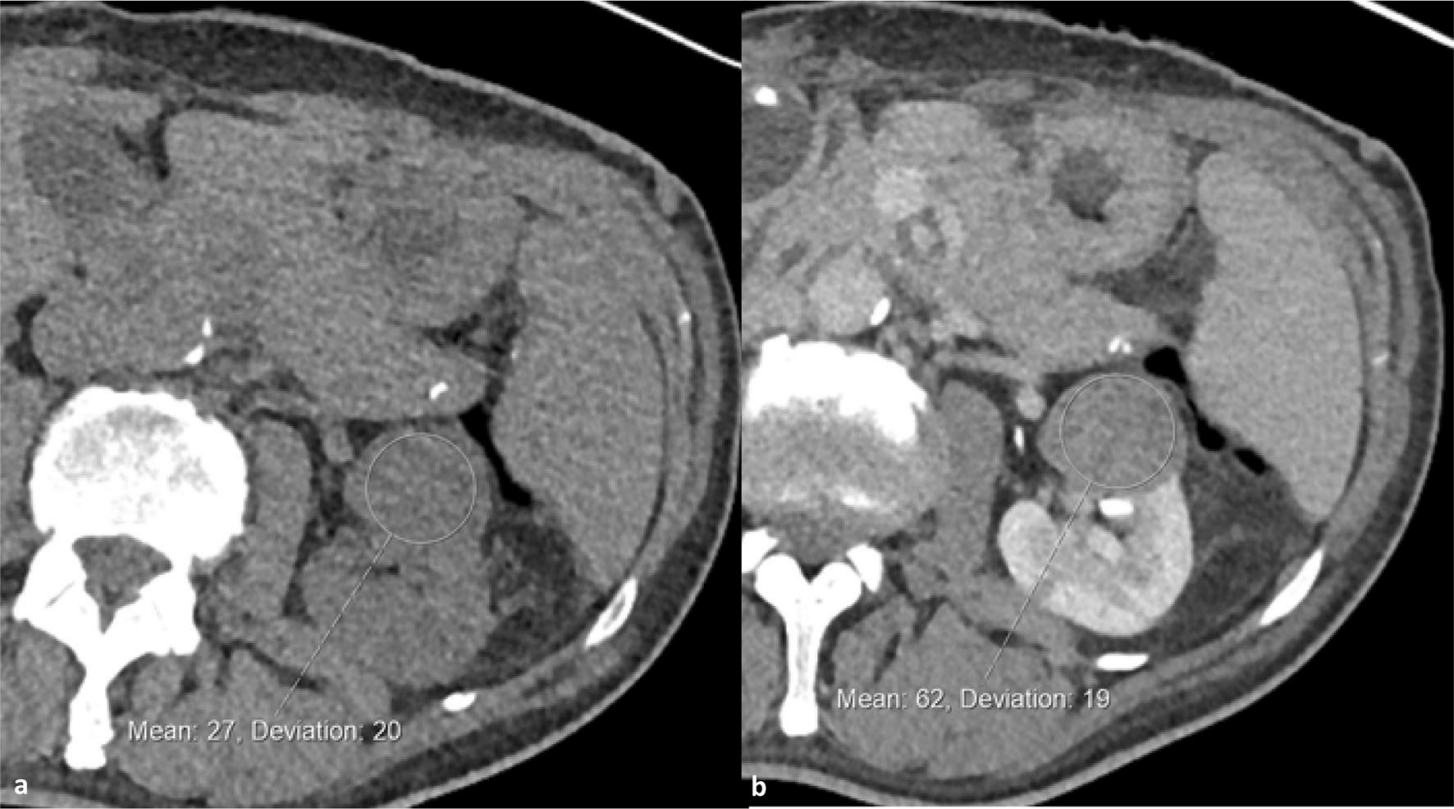
Non-enhanced (**a**) and split-bolus post-contrast nephrographic/urographic phase images (**b**) with a circular region of interest centred on a 3 cm left interpolar low attenuation renal mass demonstrating definite internal enhancement (Hounsfield units increasing from 27 to 62). This was confirmed as a type 1 papillary renal cell carcinoma

The Bosniak classification is a well-established system to classify renal cysts based on CT findings [34]. Risk of malignancy is predicted based on the appearance of the cyst wall, number and thickness of septations, calcification and enhancement (Fig. 1) [35]. Simple cysts do not enhance following contrast administration; however CT pseudo-enhancement may occur, where simple cysts appear to demonstrate enhancement < 20 Hounsfield units (HU) due to the CT reconstruction algorithm [36]. Two recent meta-analyses have demonstrated malignancy rates < 6% in Bosniak II to IIF cysts, with rates of over 50% in Bosniak III and approximately 90% in Bosniak IV lesions [35, 37]. EAU guidelines recommend surveillance for Bosniak IIF cysts and operative management for patients with Bosniak III–IV cysts, and therefore differentiating category IIF and III lesions is crucial [5]. However, considerable inter-observer disagreement has been noted in differentiating IIF and III cysts [37]. The effectiveness of the Bosniak classification system for category III cysts has been shown to be suboptimal, leading to considerable operative overtreatment in patients who are found to have benign disease (Fig. 2) [37]. Pitra et al. suggest that MRI may be used for reclassification of Bosniak IIF and III cysts, leading to significant changes in operative management in over a third of cases [38]. Magnetic resonance imaging has superior soft tissue contrast resolution compared to CT, potentially resulting in exaggerated septal thickness and more prominent septal enhancement, and may also upgrade the Bosniak classification in small (< 2.5 cm) cysts [39]. MRI also allows improved visualisation of haemorrhagic cysts, typically hyperintense on T1-weighted sequences, compared to CT [39]. Contrast-enhanced ultrasound (CEUS), involving encapsulated microbubbles of gas injected intravenously as contrast agent has shown to be as effective as CT for renal cyst classification with the Bosniak system [40], and even superior to CT for the detection of malignancy in complex renal cysts [41]. Additionally, CEUS has also been found to have a higher sensitivity and specificity compared to MRI for the differentiation of complex renal cysts and malignancies [42].

#### Solid masses

Few imaging features are discriminatory in the diagnosis of solid renal masses and approximately 20% of lesions removed at surgery will be benign as a consequence [43]. There is an increasing likelihood of malignancy and higher grade with increasing lesion size [34, 44, 45]. Of benign SRM that are surgically excised, 10–38% are found to be angiomyolipomas and 34–58% are found to be oncocytomas [46–48]. Ultrasound, contrast-enhanced CT and MRI may aid diagnosis (Table 1). The presence of enhancement, i.e. a change of ≥ 15 HU before and after contrast administration in CT, is considered the most important criterion for the differentiation of malignant solid SRM subtypes, with clear cell RCC enhancing much more compared to chromophobe and papillary RCC [5, 49]. However, reliable differentiation between RCC and oncocytoma and fat-poor angiomyolipoma remains a challenge.

**Table 1:** Rationale underlying the use of common imaging modalities in the characterisation of small renal masses

Imaging Modality	Comments
Ultrasound	User dependent Sensitivity is low for small renal lesion [26, 101] Doppler may demonstrate vascularity in the periphery of the mass, suggestive of oncocytoma No radiation, therefore ideal for repeated scanning and surveillance Does not evaluate the collecting system adequately [26]
Contrast enhanced CT	Gold standard of imaging; however, poor differentiation between solid masses, fat-poor AML and oncocytoma Enables assessment of contrast enhancement and presence of fat, two key diagnostic features [5, 49]. Presence of central stellate scar and segmental enhancement inversion is suggestive of oncocytoma Gain additional information including morphology of contralateral kidney and surgical characteristics in patients in whom surgical excision is considered
MRI	Superior soft tissue contrast resolution compared to CT May upgrade the Bosniak classification in small cysts and allows improved visualisation of haemorrhagic cysts Enables characterisation of solid renal masses No radiation burden Prone to motion artefacts Limited by MR contraindications, scanner availability and cost Useful for problem-solving, in pregnancy or when patients have an iodinated contrast allergy or renal failure
Contrast enhanced ultrasound	Involves microbubbles of gas injected intravenously as contrast agent, therefore enabling detection of slow and low flow in the microcirculation Requires a trained operator May be used for reclassification of Bosniak IIF and III cysts and to characterise solid renal masses [5]

**Fig. 3:**
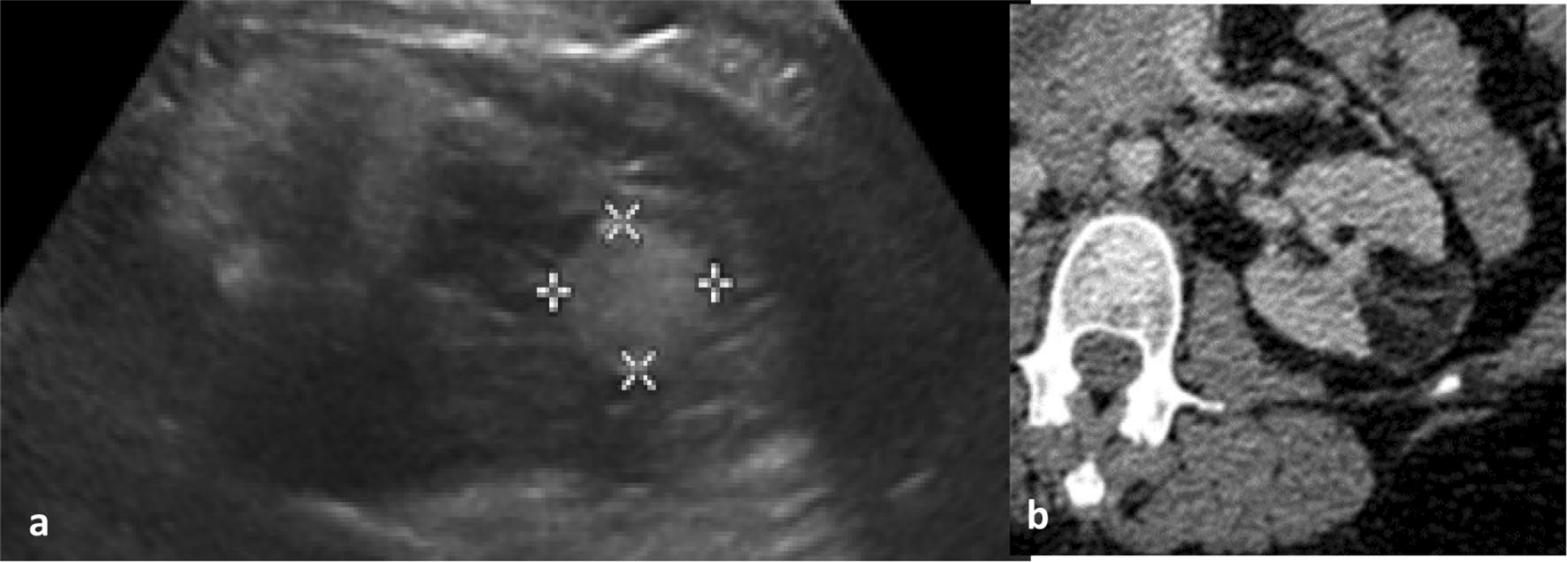
Classical ultrasound (**a**) and CT (**b**) appearances of a left renal angiomyolipoma. Solid hyperechoic mass relative to renal parenchyma and of fat attenuation on CT with enhancing components

Angiomyolipoma (AML), the most common benign renal tumour, is in most cases characterised by the presence of macroscopic fat (Fig. 3). Therefore, AMLs appear markedly hyperechoic on ultrasound and typically there is low attenuation (− 10 to − 100 HU) on unenhanced CT. On MRI, signal drop on fat-suppressed and opposed-phase T1 sequences is thought to be typical [50, 51]. Some RCCs may contain fat; however, and the presence of calcifications may point towards a diagnosis of malignancy [52]. AMLs may contain minimal fat in approximately 5% of cases and therefore may be difficult to differentiate from RCC. Fat-poor AMLs demonstrate marginally higher attenuation on unenhanced CT than RCC [52]. The combination of homogeneous enhancement and prolonged enhancement pattern on CT resulted in a positive predictive value of 91% and negative predictive value of 87% for the differentiation of fat-poor AML and RCC [50]. A meta-analysis has demonstrated a pooled sensitivity of 0.67 (95% CI 0.48–0.81) and specificity of 0.97 (95% CI 0.89–0.99) for the ability of CT to diagnose fat-poor AML [53].

Differentiating oncocytomas, the second most common benign tumour type, from RCC remains a diagnostic challenge both on imaging and renal biopsy. As a result, oncocytoma is found on 3–4% of nephrectomy pathology specimens [54]. In fact, whilst the positive predictive value of diagnosing malignancy on renal biopsy is > 99%, one in four renal biopsies reported as oncocytoma are found to be RCC following surgical excision [55]. On ultrasound, oncocytomas classically appear as well-circumscribed, homogeneous masses which may be isoechoic or hypoechoic. Oncocytomas are well vascularised; therefore, Doppler ultrasound may show increased vascularity in the periphery of the mass. On CT, these benign tumours often display homogeneous enhancement, and in the absence of calcification, necrosis and haemorrhage [56]. Oncocytomas may display a characteristic central stellate scar in up to one-third of cases, although this may be less evident in smaller lesions [57]. Chromophobe RCCs may contain a central scar, whilst other RCC subtypes that contain central necrosis may be confused for a stellate scar [58]. Segmental enhancement inversion, a tumour segment with lower signal intensity in the arterial phase and higher intensity at the early excretory phase, is considered suggestive of oncocytomas: with a high specificity (87–100%), but discrepancies in reported sensitivity [57–59].

In one study of biopsy-confirmed renal tumours, CT growth pattern, interface with the parenchyma, presence of a scar, segmental inversion of enhancement, unenhanced CT histogram and pattern of enhancement on triphasic MDCT were studied [49]. This study found that only gradual enhancement was suggestive of a benign pathology; however, it is well known that benign lesions also demonstrate early rapid enhancement, confounding interpretation [60]. For example, benign oncocytomas and malignant clear cell renal cancer share enhancement characteristics due to the dense vascularisation of oncocytomas.

Multiparametric MRI has the potential to improve the characterisation of solid renal tumours, but its diagnostic accuracy and cost-effectiveness are yet to be investigated prospectively [61, 62]. Retrospective studies have used it to differentiate small renal masses that demonstrate a high signal intensity central area on T2-weighted imaging, which may represent either oncocytoma with a central scar or RCC with central necrosis. Complete late enhancement of the central area on gadolinium-enhanced T1-weighted images, typical of fibrosis, was suggestive of oncocytoma, whereas absence of central enhancement (typical of necrosis) or presence of a signal drop on chemical shift imaging (in keeping with the presence of intracellular fat) were used to rule out oncocytoma, as it does not contain either [58]. Galmiche et al. suggest that multiparametric MRI may distinguish between oncocytoma and chromophobe RCC based on enhancement and diffusion characteristics [63]. In a study by Taouli et al., contrast-enhanced MRI performed better than diffusion MRI in differentiating between solid RCC and benign tumours (when excluding fat-rich AML), with a sensitivity of 100% and specificity of 89%; contrast-enhanced MRI combined with diffusion MRI achieved 96% specificity [61]. An important consideration is that since 2007, administration of gadolinium has been avoided in end-stage renal disease or dialysis patients due to increased risk of nephrogenic systemic fibrosis, limiting use [64].

#### Emerging technology

CEUS has been increasingly applied to differentiate small renal masses, due to its ability to detect slow and low flow in the microcirculation [5, 65, 66]. In a meta-analysis of 567 histologically confirmed RCC and 313 benign masses, the pooled sensitivity of CEUS was 88% (85–90 95% CI) and specificity was 80% (75–85% 95% CI) [65]. A recent feasibility study has demonstrated the usefulness of magnetic resonance elastography (MRE), as part of a multiparametric MR imaging protocol, to characterise 21 indeterminate SRM. MRE viscoelastic profile may discriminate between oncocytomas and clear cell RCC, and further prospective studies are warranted [67]. The emerging concept that biomedical images contain ‘hidden’ information about the underlying tissue biology that can be revealed via quantitative image analysis, an approach known as radiomics, has prompted the application of automated image analysis tools in renal tumours [68]. Early retrospective data have suggested that texture analysis of CT images combined with machine learning could distinguish RCC subtypes with an AUC > 0.90, warranting prospective investigation [69]. Molecular imaging with ^99m^technetium–sestamibi single photon emission computed tomography/computed tomography (^99m^Tc-MIBI SPECT/CT) has also been used to differentiate oncocytomas and indolent hybrid oncocytic/chromophobe tumours from other more aggressive malignant small renal tumours. The former contain numerous and dense cellular mitochondria and therefore demonstrate “hot” radiotracer uptake relative to the ipsilateral renal parenchyma, whereas the latter appear “cold” [70, 71]. Sheikhbahaei et al. evaluated the diagnostic accuracy of standard pre-operative CT, MRI and ^99m^Tc-MIBI SPECT/CT in 48 patients with small renal masses undergoing partial or radical nephrectomy. The area under the receiver operator characteristic curve for the differentiation of benign from malignant masses was 0.60 for CT and MRI alone, increasing to 0.85 with the addition of ^99m^Tc-MIBI SPECT/CT [71]. Quantitative techniques for image analysis may enable increased accuracy and use of ^99m^Tc-MIBI SPECT/CT in future [72].

### Staging

Staging in renal cancer is important for therapeutic triage and to define prognosis. Five-year survival rate is 84% in patients with Stage I (localised) cancer compared to 5% with Stage IV (metastatic) disease [73]. CT remains the first-line modality for staging of locoregional and suspected metastatic disease. Staging accuracy for CT is good with the general exception of early perinephric invasion and venous invasion. In one study of 100 pathologically proven cancers, CT correctly staged 91% of cancers [74]. The sensitivity and specificity of CT for perinephric extension, venous invasion, metastatic adenopathy and organ invasion was 46, 78, 83 and 60%, respectively, and 98, 96, 88 and 100%, respectively. The low sensitivity for perinephric invasion in this study is likely influenced by the author’s chosen definition, i.e. a 1 cm perinephric soft tissue mass, and by the use of older generation scanners. A more recent study has suggested that 9% of patients may be upstaged from T1 (organ-confined) to T3a (peri-nephric invasion) at pathology [75]. The limitation of using size for determining nodal status is well known. Up to 58% of patients with enlarged (> 1 cm short axis) nodes on CT will not have metastases [76].

**Fig. 4:**
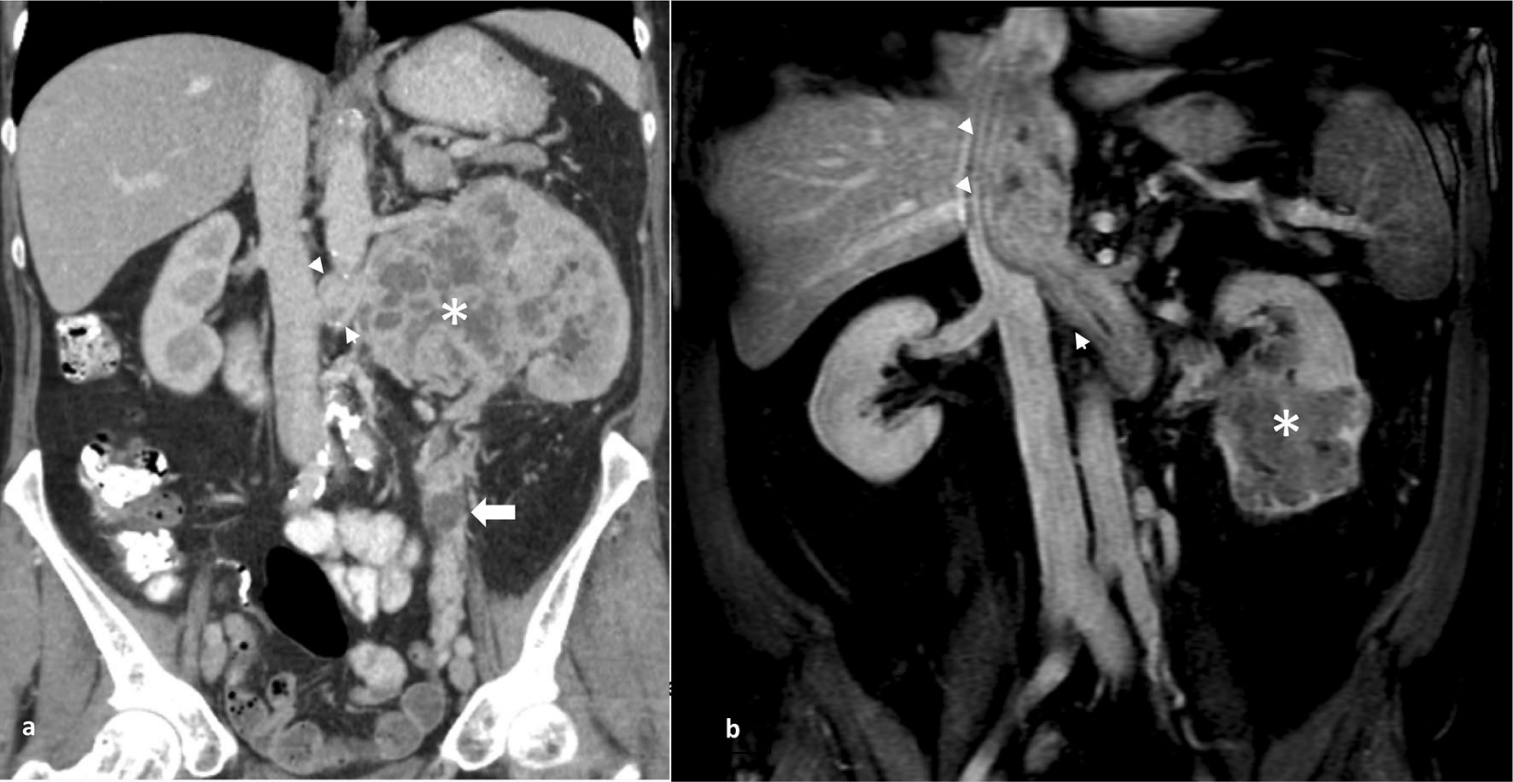
Coronal portal venous phase CT depicting a large left lower pole renal tumour (star) with direct extension into the renal vein (arrowheads) and along the gonadal vein (filled arrow). Coronal fat-saturated T1 weighted MRI images following intravenous gadolinium in a different case demonstrate enhancing tumour thrombus within the left renal vein and extending into the infradiaphragmatic vena cava (stage T3b)

MR demonstrates a greater sensitivity for inferior vena cava involvement than CT and comparable performance for perinephric invasion, metastatic adenopathy and organ invasion, but is not performed routinely (Fig. 4) [77]. Positron emission tomography (PET)/CT with ^18^F-fluorodeoxyglucose (FDG) as tracer has its limitations due to the renal excretion of FDG and thus high background activity within the kidney; however, studies have suggested comparable locoregional staging accuracy with CT alone with false negative rates of 4% [78, 79].

**Fig. 5:**
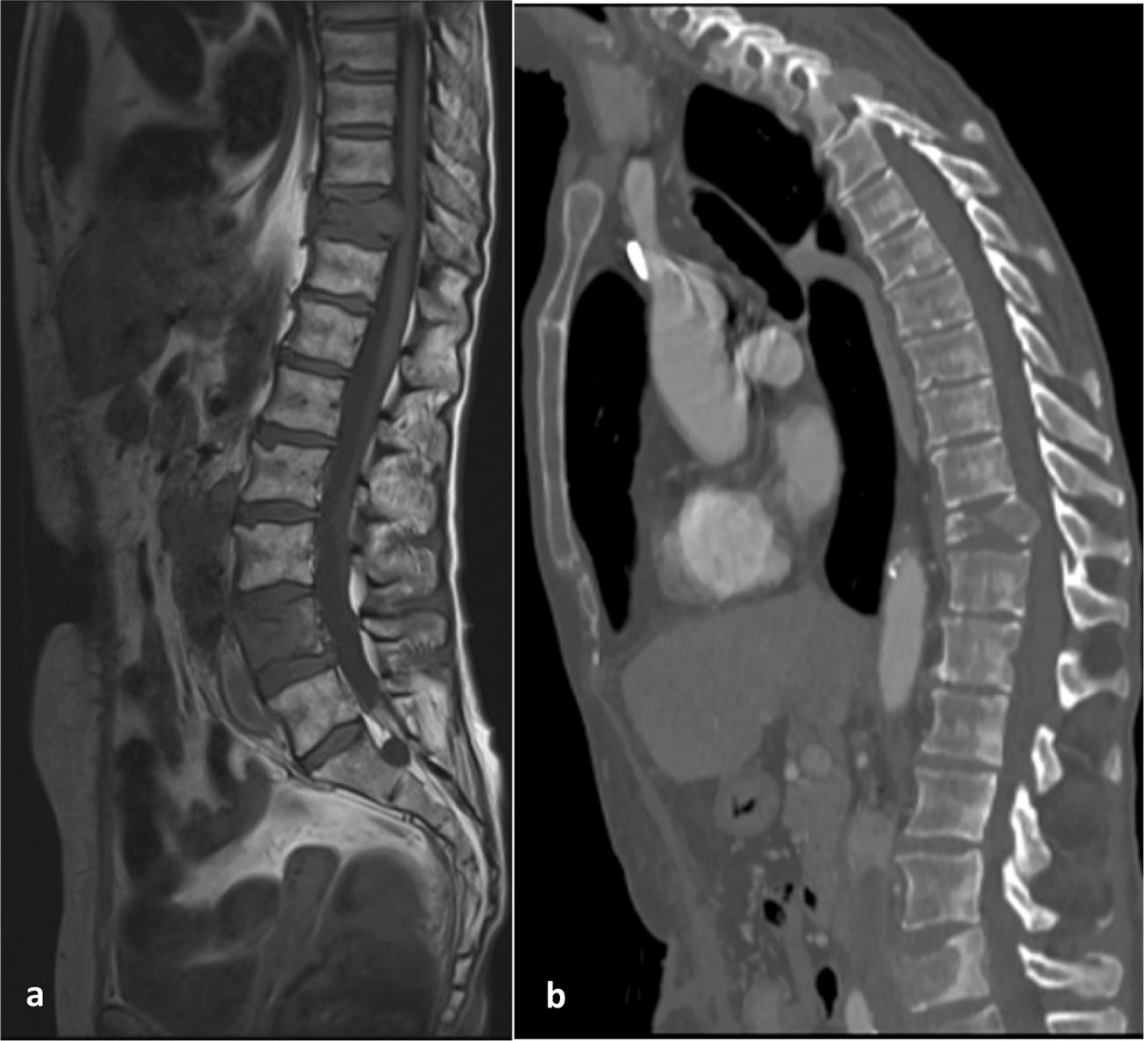
Sagittal T1 weighted MRI (**a**) and CT (**b**) images of the thoracolumbar spine demonstrating metastatic infiltration at L4 and T12 with an associated pathological fracture and narrowing of the central vertebral canal

For suspected osseous disease, bone scintigraphy (99Tc-radioisotope scan) detects areas of increased osteoblastic activity that occurs as a compensatory mechanism after bone resorption [80]. Sensitivity and specificity of 94 and 86% have been reported [81]. False negatives have been reported with bone scintigraphy due to the lytic nature of metastases. The sensitivity for bone disease may be higher with ^18^F-FDG PET/CT; however, PET/CT is not part of the standard care pathway [82]. MR is used routinely where there is clinical suspicion of spinal cord compression (Fig. 5). Whole body MR has been advocated for its higher sensitivity for the detection of bone metastases than scintigraphy, but this has not been investigated specifically in metastatic RCC; its limitations include the time taken to carry out an examination (typically, 60 min) and limited access to scanners.

### Assessment of therapy response

Percutaneous cryoablation or radiofrequency ablation of SRM is routinely performed in patients with multiple comorbidities or those wishing to preserve renal function [5–8]. However, response assessment following ablative therapy for localised RCC remains challenging, as reduction in tumour size, which is the mainstay of therapy assessment in other contexts, may not occur [12]. In fact, an increase in lesion size may be noted in the period immediately following successful ablative therapy and the pattern of enhancement may be a more reliable indicator of treatment success [83], although prediction of early recurrence remains a challenge. The hallmark of successful treatment is considered to be lack of enhancement in the treated tissue, whereas typically residual tumour or treatment failure is noted as a nodular or crescent-shaped enhancement on contrast-enhanced CT or MRI. Some studies suggest this may not be a reliable predictor, as discrepancies have been noted between enhancement on imaging and repeat biopsy results, although the accuracy of biopsy has also been called into question [84, 85]. Following radiofrequency ablation, long-term signs include a characteristic “bull’s-eye” or “halo” sign appearance [86]. A gradual reduction in lesion size may occur, but this is much more common in cryoablation than radiofrequency ablation [87, 88]. Following cryoablation, tumours may demonstrate scar formation and on occasion an enhancing rim, which may persist for several months, and is due to hyperaemia and inflammation rather than recurrence [84]. In response to a lack of uniformity in the definition of local recurrence following ablative therapy, the International Working Group on Image-Guided Tumour Ablation (2005) established the following definition: presence of localised disease remaining in the kidney, as evidenced by tumour enhancement following the first ablation, or a visible increase in lesion size at the site of the previous ablation, with or without contrast enhancement [89]. This definition was later expanded to include “the failure of an ablated lesion to regress in size over time, or the development of new satellite or port site soft tissue nodules [90, 91].”

In a series of over 600 patients treated with primary cryoablation or radiofrequency ablation, 63 patients experienced incomplete treatment (defined as residual or recurrent disease). 70% of incomplete treatments were detected within 3 months of therapy [92]. As such, AUA guidelines recommend follow-up CT or MRI with and without contrast at 3 and 6 months post-therapy, followed by annual imaging for 5 years [91]. Further research is required to establish the evidence base behind these surveillance protocols, whether they translate to a survival benefit and the associated cost to patients and society [93]. The Ablation of Renal Masses Outcomes Registry (ARMOR), which is actively recruiting patients (Clinicaltrials.gov identifier: NCT01888198) in the USA, may provide useful data in this domain. There is also a need to improve the detection of residual disease, particularly as serial biopsy may underestimate this. Further prospective study of the role of perfusion imaging in this context is warranted by early data. CEUS has emerged as a potential tool to monitor response following ablative therapy and prospective studies are currently underway (ClinicalTrials.gov Identifier: NCT01141816).

#### Response to local ablative therapy

**Fig. 6:**
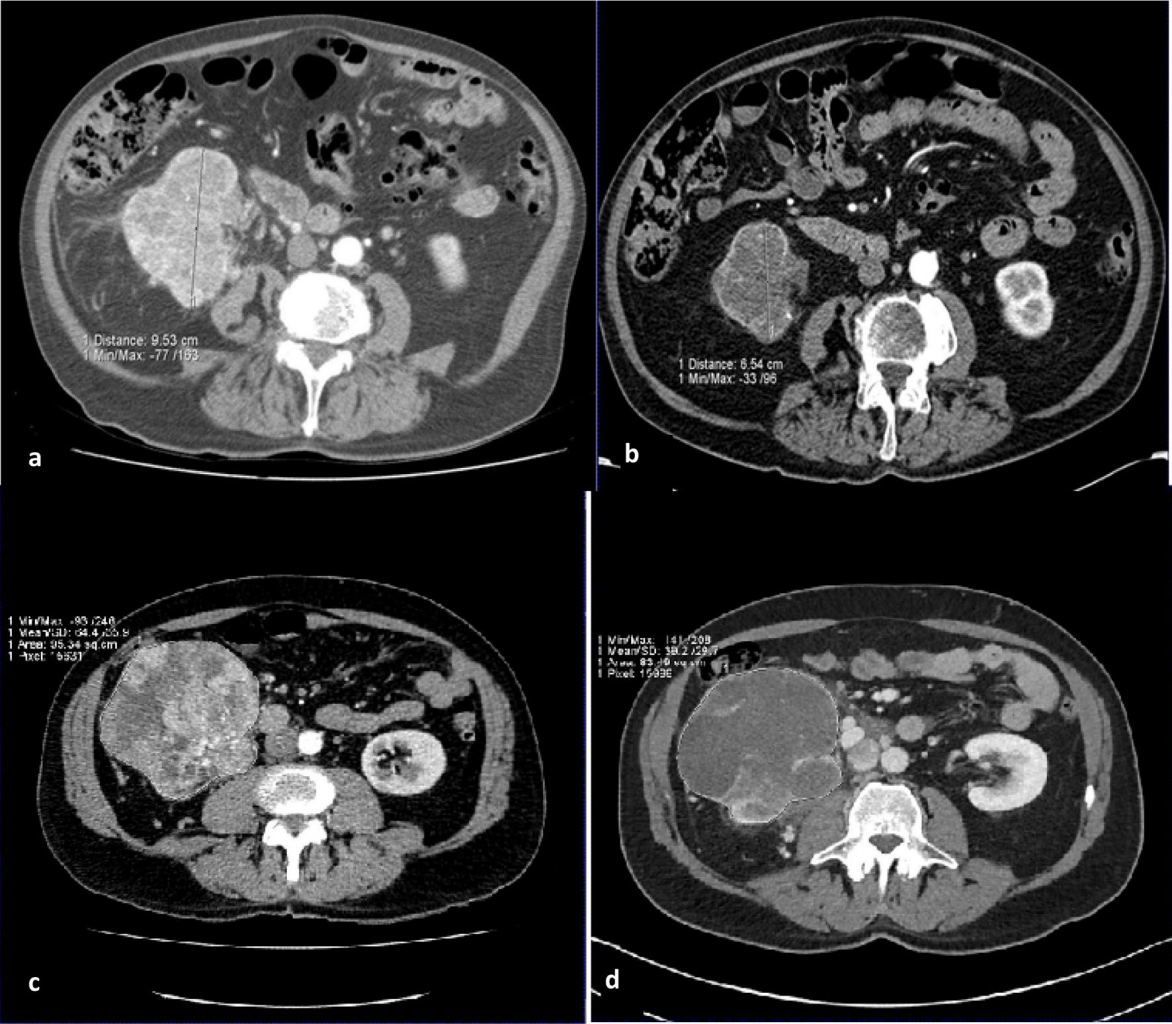
Images (**a**) baseline and (**b**) depict the 31% reduction in size of a right renal tumour over the course of 12 weeks targeted therapy, meeting criteria of partial response by RECIST v1.1. Conversely, images (**c**) at baseline and (**d**) in a different patient show that though there is clear devascularisation of the right renal primary tumour on treatment with a reduction in central enhancement, there is insufficient reduction in size to amount to a partial response by RECIST v 1.1 assessment

Similarly, challenges remain in the imaging-based response assessment of patients with metastatic RCC. An accurate assessment of response to therapy is crucial to guide clinical decisions regarding continuation of treatment. Furthermore, imaging-based progression is routinely used as a surrogate marker for survival in clinical trials; therefore, the development of standardised and validated criteria is key. Reduction in tumour size remains at the core of therapy assessment with contrast-enhanced CT. For example, response assessment is performed by measuring the serial change in tumour size in up to five target lesions for Response Evaluation Criteria in Solid Tumors (RECIST) 1.1 (Fig. 6) [12]. However, in advanced RCC, response assessment based on size change has its limitations for both anti-angiogenic and immunotherapies. Anti-angiogenic therapies have a cytostatic rather than cytotoxic mechanism of action: tumour stabilisation occurs in the majority of cases and size reduction is often less pronounced and late occurring; therefore, RECIST criteria tend to underestimate response. Some tumours may even demonstrate an early increase in size due to necrosis. As a result, a number of alternative response criteria have been proposed to assess radiologic evidence of a reduction in tumour vascularity (Table 2). Such surrogate markers include: a reduction in attenuation, evidence of necrosis (new areas of non-enhancing soft tissue) and changes in the degree and pattern of enhancement [14–16, 94, 95].

**Table 2:** Methods to assess response to anti-angiogenic therapies

Criteria	Description	Categories		
		Complete response	Partial response	Progressive disease
Response Evaluation Criteria in Solid Tumors (RECIST) version 1.1 (2009) [12]	Assess reduction in target lesion size, where size is defined as the sum of the maximum axial diameter length of each lesion (up to 2 lesions in the same organ, up to 5 in total). All other metastases are considered non-target lesions	Complete resolution of all lesions	Reduction in size ≥ 30% of target lesions, no progression and no new lesions	New lesions or increase in size ≥ 20%
Choi Criteria (2007) [13]	Originally developed to evaluate the response of gastrointestinal stromal tumours to imatinib, a tyrosine kinase inhibitor Assess reduction in target lesion size or arterial phase density	Complete resolution of all lesions	Reduction in size ≥ 10% or reduction in mean attenuation ≥ 15%, no progression and no new lesions	New lesion or increase in size ≥ 10%
Modified Choi Criteria (2010) [14]	Assess reduction in target lesion size and arterial phase density combined	Complete resolution of all lesions	Reduction in size ≥ 10% and reduction in mean attenuation ≥ 15%, no progression and no new lesions	New lesion or increase in size ≥ 10%

Criteria	Description	Categories		
		Favourable response	Indeterminate response	Unfavourable disease
Size and Attenuation CT (SACT) [15]	Assess reduction in size of target lesions or volumetric mean tumour attenuation	No new lesions and reduction in size ≥ 20% or reduction in size ≥ 10% and reduction in mean attenuation ≥ 20 HU in ≥ 50% of non-pulmonary target lesions or reduction in mean attenuation ≥ 40 HU in at least one non-pulmonary target lesion	Response which does not fit other categories	Increase in size ≥ 20% or new lesion, MCF of an existing target lesion, or new enhancement in a homogeneously hypoattenuating non-enhancing lesion
Morphology, Attenuation, Size, and Structure (MASS) Criteria (2010) [16]	Assess morphology, attenuation, size, and structure of target lesions including an assessment of marked central necrosis (MCN) and marked central fill in (MCF) MCN is defined as a change to near fluid attenuation in ≥ 50% of the enhancing central portion of a predominantly solid enhancing mass (subjective assessment). MCF is defined as a change from central necrosis to almost complete central enhancement in a solid mass on contrast enhanced CT (subjective assessment)	No new lesions and reduction in size ≥ 20% or predominantly solid enhancing lesions with MCN or reduction in mean attenuation ≥ 40 HU		Increase in size ≥ 20% in the absence of MCN or decrease in attenuation or new lesion, MCF of an existing target lesion, or new enhancement in a homogeneously hypoattenuating non-enhancing lesion

Additionally, a number of emerging techniques have been investigated. CT texture analysis using an image processing algorithm to assess heterogeneity in tumour morphology has been proposed as a marker of response [96]. Functional imaging has been investigated, including dynamic contrast-enhanced (DCE) CT, DCE-MRI, DCE-ultrasound and PET [97]. DCE imaging follows the bio-distribution of a contrast agent injected intravenously and then absorbed into the tumour microcirculation, providing information on the tumour microenvironment and vascularity pre- and post-anti-angiogenic therapy [94]. A radiomic approach synthesising results from a combination of imaging modalities may play a role in future [97]. Recently, Crusz et al. noted that in a group of 27 patients with metastatic RCC receiving tyrosine kinase inhibitors, over 50% of individuals demonstrated heterogeneous responses to therapy (defined as lesions in at least two of the following three response categories: responding, progressing and stable), potentially reflecting molecular intra-tumoral heterogeneity [98]. This may have important implications for clinical decision making, such as the choice to continue therapy in individuals with heterogeneous responses.

Due to their targeted mechanism of action, eliciting an immune response, immunotherapies have been known to lead to “pseudoprogression” or “tumour flare,” with consequent underestimation of the overall survival benefits using the traditional RECIST 1.1 criteria [18]. Partly because the pathophysiological mechanism underlying pseudoprogression is incompletely understood, differentiating this phenomenon from true progression represents an imaging diagnostic challenge with important clinical implications [99]. Four patterns of response to immunotherapy have been described: (1) an initial transient increase in target lesion size followed by durable response; (2) the development of new lesions followed by durable response in target lesions; (3) reduction in target lesion size from the outset; and (4) initially no change in target lesion size followed by a slow reduction in size [17]. To capture this response heterogeneity and variability, the ‘Immune-Related Response Criteria’ (irRC) were first developed, based on a bi-dimensional assessment of target lesion size (Table 3). In contrast to RECIST 1.1, the development of new lesions in irRC is not automatically considered as evidence of progression; rather, the size of the new lesion is added to the total tumour burden. Following irRC, a modified version of RECIST 1.1 for immune-based therapeutics (iRECIST) was proposed, returning to unidimensional assessment of lesions. The main novelty in iRECIST criteria is the differentiation between unconfirmed progressive disease (iUPD) and confirmed progressive disease (iCPD), to reflect the fact that iUPD may indeed represent pseudoprogression [100]. Most recently in 2018, the Immune-modified RECIST (imRECIST) criteria were published, once again utilising unidimensional measurements to improve reproducibility (by reducing measurement variability and error inherent in acquiring two dimensions per lesion). All these sets of criteria require prospective validation. Tumour response patterns may even vary between specific immunotherapy agents, and between tumour types: further response criteria modifications may be inevitable in the future [18].

**Table 3:** Methods to assess response to immunotherapies

Criteria	Description	Categories		
		Complete response	Partial response	Progressive disease
Response Evaluation Criteria in Solid Tumors (RECIST) version 1.1 (2009) [12]	Assess reduction in unidimensional target lesion size, where size is defined as the sum of the maximum axial diameter length of each lesion (up to 2 lesions in the same organ, up to 5 in total). All other metastases are considered non-target lesions	Complete resolution of all lesions	Reduction in size ≥ 30% of target lesions compared to baseline, no progression and no new lesions	New lesions OR increase in size ≥ 20% compared to nadir and absolute increase in size ≥ 5 mm
Immune-related response criteria (irRC) [17]	Originally developed based on response to ipilimumab in patients with melanoma Assess bi-dimensional target lesion size at baseline, where size is defined as the sum of the products of the two largest perpendicular diameters (SPD) of each target lesion (up to 5 lesions in the same organ, up to 10 in total) The total tumour burden is defined as sum of the target lesion size (calculated as above using the SPD) and the size of measurable new lesions (calculated using the SPD; where each measurable lesion must ≥ 5 × 5 mm in size)	Complete resolution of all lesions, confirmed on consecutive imaging ≥ 4 weeks apart	Reduction in tumour burden ≥ 50% compared to baseline, confirmed on consecutive imaging ≥ 4 weeks apart	Increase in tumour burden ≥ 25% compared to nadir, confirmed on consecutive imaging ≥ 4 weeks apart

Criteria	Description	Categories		
		Immune complete response (iCR)	Immune partial response	Immune progressive disease
Immune-modified RECIST (imRECIST) (2018) [18]	Assess reduction in unidimensional target lesion size, where size is defined as the sum of the maximum axial diameter length of each lesion (up to 2 lesions in the same organ, up to 5 in total). The total tumour burden is defined as sum of the target lesion size (calculated as above) and the size of measurable new lesions (maximum 2 new measurable target lesions per organ, 5 in total)	Complete resolution of all target and non-target lesions	Reduction in total tumour burden ≥ 30% compared to baseline	Increase in total tumour burden ≥ 20% compared to nadir and absolute increase in size ≥ 5 mm

## Response to therapy for metastatic disease

Imaging plays a key role throughout the RCC patient pathway, from diagnosis and staging of the disease, to the assessment of response to therapy. Characterisation of incidentally detected small renal masses remains a diagnostic challenge due to the overlap in morphological and physiological characteristics of malignant and benign lesions. A multiparametric imaging approach is most likely to yield the highest diagnostic accuracy. CT remains an accurate imaging modality for staging renal cancer: its previously documented limitations in detecting early perinephric and venous extension warrant further investigation with state-of-the-art scanners. In this context, locoregional staging may be improved with state-of-the-art MRI, including diffusion-weighted imaging. Accurate response assessment following therapy is key to guide management and inform patients about their prognosis and treatment. There is a need to improve the detection of residual disease following ablative therapy, and several clinical studies are currently underway in this field. In the era of targeted therapies and immunotherapy, size-based response assessment is limited: further development and validation of targeted imaging techniques and of response criteria that better reflect the effect of these agents are required for metastatic renal cell cancer.
